# Cost, health impacts and cost effectiveness of iceless refrigeration in India's last-mile vaccine cold chain delivery

**DOI:** 10.1093/trstmh/trac115

**Published:** 2022-12-20

**Authors:** Katherine Plewes, Panarasri Khonputsa, Nicholas P J Day, Yoel Lubell

**Affiliations:** Mahidol Oxford Tropical Medicine Research Unit, Mahidol University, 10400, Bangkok, Thailand; Centre for Tropical Medicine and Global Health, Nuffield Department of Clinical Medicine, University of Oxford, Oxford, OX3 7LF, UK; Department of Medicine, University of British Columbia, V5Z 3J5, Vancouver, Canada; Mahidol Oxford Tropical Medicine Research Unit, Mahidol University, 10400, Bangkok, Thailand; Mahidol Oxford Tropical Medicine Research Unit, Mahidol University, 10400, Bangkok, Thailand; Centre for Tropical Medicine and Global Health, Nuffield Department of Clinical Medicine, University of Oxford, Oxford, OX3 7LF, UK; Mahidol Oxford Tropical Medicine Research Unit, Mahidol University, 10400, Bangkok, Thailand; Centre for Tropical Medicine and Global Health, Nuffield Department of Clinical Medicine, University of Oxford, Oxford, OX3 7LF, UK

**Keywords:** cold chain, cost-benefit analysis, ice-less vaccine carrier, vaccine transport

## Abstract

**Background:**

Compared with ice-based vaccine carriers (IBVCs), iceless vaccine carrier (ILVC) last-mile delivery could optimize vaccine effectiveness by reducing spoilage. We estimated ILVC-associated spoilage costs averted and cost effectiveness.

**Methods:**

IBVC vaccine spoilage costs were estimated for six vaccines. ILVC incremental costs were based on yearly ILVC cost over total doses. Cost effectiveness was estimated via Markov modeling of rotavirus vaccine.

**Results:**

The spoilage cost using IBVCs was US$9 603 294. Using ILVCs, the incremental cost per vaccine dose was US$0.026, the cost-benefit ratio was 0.28, the number of averted disability-adjusted life years was 0.03 per child and there was a saving of US$0.80 per child vaccinated.

**Conclusions:**

ILVCs may bring cost savings and health gains compared with IBVCs.

## Introduction

Last-mile immunization cold chain delivery reliant on ice-based vaccine carriers (IBVCs) faces problems of accidental freezing and/or warming, as well as a lack of temperature monitoring. Approximately 65% of diphtheria, pertussis and tetanus vaccine vials showed evidence of freezing in vaccine stores and peripheral health facilities across 10 states in India.^1^ Damaged vaccines due to cold chain failings result in increased costs and potential harm; if damaged vials are administered, a blunted antibody response increases the risk of contracting vaccine-preventable diseases.

We present estimated spoilage cost in IBVC and spoilage costs averted in portable iceless temperature-regulated vaccine carrier (ILVC) cold chains, incremental cost per vaccine dose delivered using ILVCs and modeled health gains and cost effectiveness of ILVCs for last-mile vaccine delivery of rotavirus vaccine in rural India.

## Materials and Methods

Costs of spoilage due to IBVCs and ILVCs were calculated by: number of vaccine doses*cost per dose*number of eligible people*vaccine coverage*vaccine spoilage (%). The costs per dose were estimated first excluding and then including a program cost of US$0.25. Vaccine spoilage using IBVCs and ILVCs were estimated at 25% and 10%, respectively. The 2015 estimates of the number of children and women in India who were eligible, and the number of doses for each vaccine specific to these populations according to the Expanded Programme on Immunization (EPI) schedule were used (Supplementary Table S1); the rural population was estimated at 68%.

The incremental cost of ILVCs was calculated as the yearly ILVC cost/number of eligible population per year. It was estimated that a health centre (HC) would need one ILVC, at an annual cost of US$475. HCs are the last cold chain point receiving vaccines from (sub)district vaccine stores; IBVCs are used to distribute from HCs to vaccination session sites. In 2015, there were 25 555 HCs in India. The average number of each eligible population type per HC was calculated as the number of eligible population/number of HCs.

The cost effectiveness of ILVCs for rotavirus vaccine was estimated using a simplified Markov model validated by comparison with a published model.^2^ The model included three health states (well, symptomatic rotavirus gastroenteritis and dead). The model incidence of rotavirus gastroenteritis was fit using data from India showing an annual risk of 8394/100 000 in children aged <5 y.^3^ The benefit of an ILVC to reduce spoilage was then modeled. Supplementary Table S2 shows the key parameter estimates. One-way sensitivity analysis for the incremental cost of an ILVC per vaccine and their associated spoilage was performed.

Disability-adjusted life years (DALYs) per case of gastroenteritis were estimated by combining a disability weight of 0.1 and an assumed duration of 1 week per case, with years of life lost resulting from rotavirus gastroenteritis-associated deaths calculated using the probability of severe disease*mortality in severe disease*a life expectancy of 63 y (Supplementary Table S2).

## Results

The costs of spoilage using an IBVC cold chain system was estimated to be US$9 603 294 based on the number of children and women in rural India eligible for routine EPI vaccinations (Supplementary Tables S1 and 9). Spoilage costs increased significantly when program costs were included.

The incremental cost per vaccine dose delivered with an ILVC was estimated to be US$0.026. On average, an HC serves a population of 6223 for routine vaccination, delivering approximately 18 487 vaccine doses. The total cost of an ILVC per 5 y of use was US$2 375 (US$475 annually) (Supplementary Table S4). This compared with annual vaccine spoilage of US$1 726 per HC. Therefore, the cost-benefit ratio for ILVC that avoided this wastage was 0.28, indicating that ILVCs are cost beneficial.

**Figure 1.**
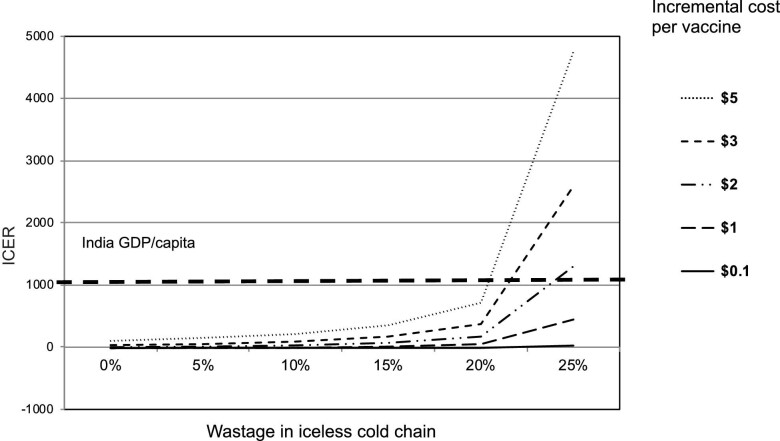
Incremental costs per vaccine and vaccine spoilage using a portable iceless, temperature-regulated vaccine carrier for last-mile cold chain vaccine delivery. ICER, incremental cost-effectiveness ratio.

Using the current IBVCs, vaccines were cost effective with a cost per DALY averted of US$216, slightly higher than prior estimates due to the lower incidence in our model. Switching to an ILVC would avert a further 0.03 DALYs per child with cost savings of US$0.80 per child vaccinated. The sensitivity analysis suggested that even at a higher incremental delivery cost of US$2.00 per vaccinated child and substituting higher spoilage (up to 20%) using the ILVC (compared with 25% using an IBVC), the ILVC is still cost effective (Figure 1).

## Discussion

Vaccine spoilage using IBVCs incurs high human and economic costs, whereas the per dose incremental delivery cost using ILVCs to reduce or eliminate such spoilage is negligible. In a scenario where spoiled vaccines are identified and replaced, ILVC use could result in large cost savings. Further, in a scenario where spoiled vaccines are administered, an ILVC could be cost saving and provide health gains. These findings are robust to variation in the incremental cost of vaccine delivery using ILVCs and more modest gains in spoilage avoided.

## Conclusions

ILVCs represent a potential vital and cost-effective disruptive technology that could improve the efficiency of immunization programs, particularly in Brazil, Russia, India, China and South Africa (BRICS) nations whose health expenditures are projected to increase until 2030 and require healthcare policies factoring in resource allocation.^4^

## Supplementary Material

trac115_Supplemental_File

## Data Availability

The data that support the findings of this study are available online and from the corresponding author upon reasonable request.
