# Supplementary material for: Cost, health impacts and cost effectiveness of iceless refrigeration in India's last-mile vaccine cold chain delivery
Source: Trans R Soc Trop Med Hyg. 2022 Dec 20;117(4):310–2. doi: 10.1093/trstmh/trac115 (PMC10069297; doi:10.1093/trstmh/trac115)
Supplement: trac115_Supplemental_File [file trac115_supplemental_file.pdf]

Web extra material

Supplementary Appendix: This appendix has been provided by the authors to communicate additional detail of their work.

Cost, health impacts and cost-effectiveness of iceless refrigeration in India's last-mile vaccine cold chain delivery

Katherine Plewes, Panarasri Khonputsa, Nicholas P.J. Day, Yoel Lubell

Table of Contents

Supplementary tables 3
References 5
WHO EPI Fact Sheet 6

Supplementary tables

Table S1. Number of children and women eligible for routine vaccination in rural India in 2015

Target Population	Number of vaccine doses per vaccine required per demographic*						Number of eligible children and women living in rural areas[a]
	BCG	DPT	TT	Hepatitis B	OPV	Measles	
At birth	1	-	-	1	1	-	18 645 600
Age in 1 year	-	-	-	-	3	1	17 897 736
Age in 5 years	-	2	-	-	1	1	63 362 264
Women (pregnant)	-	-	2	-	-	-	20 508 800
Women (child-bearing age)	-	-	1	-	-	-	163 964 320

Abbreviations: BCG, Bacillus Calmette-Guérin vaccine; DPT, Diphtheria, pertussis, and tetanus vaccine; OPV, Oral polio vaccine; TT, Tetanus toxoid vaccine.
* http://www.searo.who.int/entity/immunization/data/india.pdf?ua=1 - World Health Organization. (2016). EPI Fact Sheet India, online reference removed; included in Supplementary file.
[a] = total number of eligible women and children × proportion living in rural area (68%)

Table S2. Parameter estimates included in cost effectiveness analysis using simplified Markov model

Key parameters	Estimate
Incidence of rotavirus gastroenteritis[1]	0.29*
Probability of episode being severe[2]	0.28**
Mortality in severe cases[2]	0.068
Protective effect of vaccine[3] (%)	48
Disability weight per case of gastroenteritis[4]	0.1***
Vaccine spoilage using ice-based vaccine carrier cold chain (%)	25
Vaccine spoilage using iceless-based vaccine carrier cold chain (%)	10
Incremental cost per vaccinated child for iceless vaccine carrier cold chain (US$)	0.10

*The model was fit to the reported rate of 8,394/100,000 in children < 5 years of age using this incidence at 6 months and declining exponentially with age. **At 6 months, declines exponentially with age reaching <1% at 30 months.
***Between the disability weight for mild diarrheal disease of 0.074 and 0.188 for moderate diarrheal disease.
[1] John J, Sarkar R, Muliyil J et al. Rotavirus gastroenteritis in India, 2011-2013: revised estimates of disease burden and potential impact of vaccines. Vaccine 2014;32 Suppl 1:A5-9.
[2] Rose J, Parashar UD. Should India launch a national immunisation programme against rotavirus? Yes. BMJ 2012;345:e7818.
[3] Soares-Weiser K, Bergman H, Henschke N et al. Vaccines for preventing rotavirus diarrhoea: vaccines in use. Cochrane Database Syst Rev 2019;2019:CD008521.
[4] Global Burden of Disease Collaborative Network. Global Burden of Disease Study 2019 (GBD 2019) Disability Weights. Seattle, United States of America: Institute for Health Metrics and Evaluation (IHME), 2020. https://doi.org/10.6069/1W19-VX76

Table S3. Vaccine cost per dose, spoilage, coverage and costs or spoilage using ice-based vaccine carrier

Vaccine Type	Cost per dose[5] (US$)	Spoilage at session site using ice-based vaccine carrier (%)	Current vaccine coverage[5] (%)	Avoidable cost of vaccine spoilage (US$)[a]	Avoidable cost of vaccine spoilage and program cost (US$)[b*]
BCG	0.05	25	95	221 417	1 328 499
DPT	0.04	25	100	1 267 245	9 187 528
TT	0.02	25	85	2 961 718	39 983 196
Hepatitis B	0.05	25	100	233 070	1 398 420
OPV	0.06	25	90	1 831 964	9 465 150
Measles	0.16	25	95	3 087 880	7 912 693
**Total**				**9 603 294**	**69 275 486**

Abbreviations: BCG, Bacillus Calmette-Guérin vaccine; DPT, Diphtheria, pertussis, and tetanus vaccine; OPV, Oral polio vaccine; TT, Tetanus toxoid vaccine.

[a] = cost per dose * number of required doses * spoilage * vaccine coverage * eligible people

[b]= cost of vaccine and program per dose * number of required doses * spoilage * vaccine coverage * eligible people

*Includes 0.25 USD program costs per dose

[5] Chatterjee S, Pant M, Haldar P et al. Current costs & projected financial needs of India's Universal Immunization Programme. Indian J Med Res 2016;143:801–8.

Table S4. Incremental cost of vaccine delivery using iceless vaccine carrier

**Target population**	**Individuals per health centre**	**Doses per vaccine**	**Subtotal number of doses**	**Cost of iceless vaccine carrier (US$)**	
At birth	730	3	2 189	Unit cost[a]	2 000
In 1 year	679	4	2 717	5-year cost[b]	375
In 5 years	2405	5	12 024	Cost per year[c]	475
Pregnant women	778	2	1 557		
Total	6 223	--	18 487	Cost per dose	0.026

[a] 5-year estimated shelf life of iceless vaccine carrier

[b] 5-year maintenance cost

[c] = (unit cost + maintenance cost)/5

References

1. John J, Sarkar R, Muliyil J *et al.* Rotavirus gastroenteritis in India, 2011-2013: revised estimates of disease burden and potential impact of vaccines. *Vaccine* 2014;**32 Suppl 1**:A5-9.

2. Rose J, Parashar UD. Should India launch a national immunisation programme against rotavirus? Yes. *BMJ* 2012;**345**:e7818.

3. Soares-Weiser K, Bergman H, Henschke N *et al.* Vaccines for preventing rotavirus diarrhoea: vaccines in use. *Cochrane Database Syst Rev* 2019;**2019**:CD008521.

4. Global Burden of Disease Collaborative Network. Global Burden of Disease Study 2019 (GBD 2019) Disability Weights. 2020, DOI: 10.6069/1W19-VX76.

5. Chatterjee S, Pant M, Haldar P *et al.* Current costs & projected financial needs of India's Universal Immunization Programme. *Indian J Med Res* 2016;**143**:801–8.

WHO EPI Fact Sheet

World Health Organization
Regional Office for South-East Asia

EPI Fact Sheet

India **2016**

Immunization system highlights

- There is a comprehensive multi-year plan (cMYP) for immunization covering 2013-2017.
- National technical advisory group on immunization (NTAGI) fully functional.
- A national system to monitor adverse events following immunization (AEFI) exists.
- A national policy for health care waste management including waste from immunization activities exists.
- 39% spending on vaccines financed by the government.
- 42% spending on routine immunization programme financed by the government.
- Of 649 districts (data available), 443 (68%) districts had ≥80% coverage for DTP-Hib-HepB3.
- Of 638 districts (data available), 242 (38%) districts had ≥90% coverage for MCV1.
- No stock-out of vaccines reported in 2015.
- Introduced polio vaccination policy for travellers to and from polio endemic/infected countries in 2014.
- Mission Indradhanush to immunize all children against seven VPDs ongoing since December 2014.
- Plan to introduce Rubella vaccine in 2016-2017.

Source: WHO/UNICEF joint reporting form (JRF) 2015

Table 1: Basic information[1] 2015

Total population	1 281 150 000
Live births (LB)	27 420 000
Children <1 year	26 320 200
Children <5 years	119 500 000
Children <15 years	394 400 000
Pregnant women	30 160 000
Women of child bearing age (15-49 years)	241 240 000
Neonatal mortality rate	29.2 (per 1,000 LB)
Infant mortality rate	41.4 (per 1,000 LB)
Under-five mortality rate	52.7 (per 1,000 LB)
Maternal mortality ratio	190 (per 100,000 LB)

Division/Province/State/Region	36
District	675
Block	5958
Population density (per sq. km)	382
Population living in urban areas	32%
Population using improved drinking-water sources	93%
Population using improved sanitation	36%
Total expenditure on health as % of GDP	3.8%
Births attended by skilled health personnel[2]	67%
Neonates protected at birth against NT	87%

[1] *SEAR annual EPI reporting form, 2015 and WHO, World Health Statistics 2015*
[2] *Institutional births (WHS, 2015)*

Table 2: Immunization schedule, 2015

Vaccine	Age of administration
BCG	At birth
HepB	At birth
OPV	At birth, 6th, 10th, 14th weeks and 16-24 months
IPV	14th weeks
DTP-Hib-HepB	6th, 10th, 14th weeks
DTP	16-24 months and 5 years
Measles	9 months and 16-24 months
JE	9-12 months and 16-24 months (JE endemic districts)
TT	10 years, 16 years and 2 doses/ booster for pregnant women
Vitamin A	9 months, 18 months and 6 months interval till age 5 years

Source: WHO/UNICEF joint reporting form (JRF) 2015

EPI history

- EPI launched in 1978 with DPT, OPV, BCG and typhoid vaccines.
- TT immunization of pregnant women introduced in 1983.
- Measles vaccine introduced in 1985.
- HepB piloted in 2002 and made universal in 2011.
- Second dose of measles containing vaccine introduced 2010 onward.
- Hib Pentavalent (DTP-Hib-HepB) introduced in two states in 2011 and gradually expanded to all states by 2015.
- Two doses of JE (9-12 months and 16-24 months) introduced in 2013 in endemic districts.
- Multi-dose vial policy for vaccines introduced in 2013.
- IPV introduced in six states in 2015 and being expanded to all states in 2016.
- Rotavirus vaccine introduced in four states in 2016.
- Type 2 component of OPV withdrawn on 25 April 2016 by switching from tOPV to bOPV.

Source: cMYP 2013-2017 and EPI/MOHFW

Disclaimer: The boundaries and names shown and the designations used on all the maps do not imply the expression of any opinion whatsoever on the part of the World Health Organization concerning the legal status of any country, territory, city or area or of its authorities, or concerning the delimitation of its frontiers or boundaries.

World Health Organization• SEARO/FGL/IVD • 31 August 2016

Immunization and Vaccine Development South-East Asia Region

Figure 1: National immunization coverage, 1980–2015

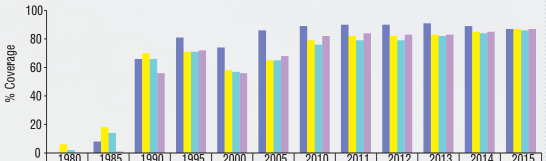

	1980	1985	1990	1995	2000	2005	2010	2011	2012	2013	2014	2015
BCG	0	8	66	81	74	86	89	90	90	91	89	87
DTP3	6	18	70	71	58	65	79	82	82	83	85	87
OPV3	2	14	66	71	57	65	76	79	79	82	84	86
MCV1	0	1	56	72	56	68	82	84	83	83	85	87

Source: WHO and UNICEF estimates of national immunization coverage, July 2016 revision

Figure 2: DTP3 coverage[1], diphtheria and pertussis cases[2], 1980–2015

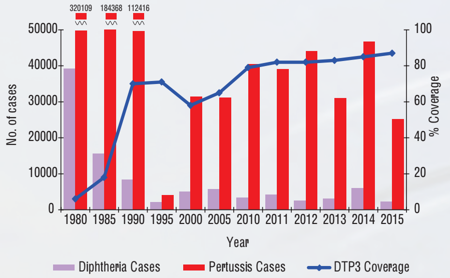

[1] WHO and UNICEF estimates of national immunization coverage, July 2016 revision
[2] WHO vaccine-preventable diseases: monitoring system 2016

Figure 3: DTP-Hib-HepB3 coverage, 2015

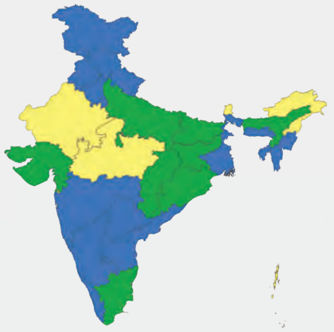

Source: SEAR annual EPI reporting form, 2015 (administrative data)

Figure 4: TT2+ coverage[1] and NT cases[2], 1980–2015

India achieved maternal neonatal tetanus (MNT) elimination status in 2015.

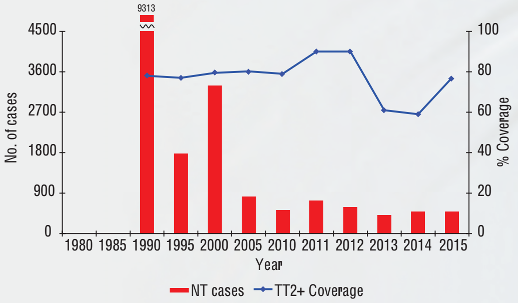

[1] Country official estimates, 1980–2015
[2] WHO vaccine-preventable diseases: monitoring system 2016

Table 3: OPV supplementary immunization activities (SIA), 2012–2015

Start date	Activity	Children immunized (<5 years)	Coverage based on independent monitoring (%)	Start date	Activity	Children immunized (<5 years)	Coverage based on independent monitoring (%)
15-Jan-12	SNID	28 077 574	98	19-Jan-14	NID	167 829 448	98
19-Feb-12	NID	168 692 708	97	23-Feb-14	NID	168 408 571	97
18-Mar-12	SNID	55 782 790	98	06-Apr-14	SNID	46 879 086	99
15-Apr-12	NID	168 211 425	97	21-Sep-14	SNID	70 228 928	99
17-Jun-12	SNID	70 890 419	98	16-Nov-14	SNID	71 564 828	99
9-Sep-12	SNID	70 365 941	98	18-Jan-15	NID	167 393 125	97
4-Nov-12	SNID	70 455 848	99	22-Feb-15	NID	168 041 923	97
20-Jan-13	NID	167 472 681	97	26-Apr-15	SNID	71 249 633	99
24-Feb-13	NID	168 152 003	97	21-Jun-15	SNID	49 182 923	98
07-Apr-13	SNID	70 746 314	98	13-Sep-15	SNID	22 481 343	98
16-Jun-13	SNID	70 312 783	98	22-Nov-15	SNID	21 594 303	98
15-Sep-13	SNID	70 308 301	98	13-Dec-15	Mop-up	6 441 536	97
24-Nov-13	SNID	70 782 906	98	–	–	–	–

Source: WHO/UNICEF JRF and EPI/MOHFW

Table 4: AFP surveillance performance indicators, 2006–2015

The last polio case due to wild polio virus (WPV) was reported on 13 January 2011 from West Bengal.

Indicator	2006	2007	2008	2009	2010	2011	2012	2013	2014	2015
AFP cases	32 194	41 524	45 585	50 405	55 785	60 540	60 922	54 660	54 101	46 978
Wild poliovirus confirmed cases	676	874	559	741	42	1	0	0	0	0
Compatible cases	494	447	539	473	190	54	31	33	13	11
AFP rate	7.63	9.71	10.50	11.64	12.70	13.51	13.95	12.51	12.52	10.78
Non-polio AFP rate[1]	7.35	9.40	10.25	11.35	12.65	13.49	13.94	12.50	12.37	10.78
Adequate stool specimen collection percentage[2]	82%	84%	84%	83%	83%	84%	87%	86%	87%	86%
Total stool samples collected	62 649	80 614	88 316	97 648	108 107	117 774	119 329	110 420	105 939	91 868
% NPEV isolation	21	20	23	22	21	20	16	19	18	15
% Timeliness of primary result reported[3]	99	98	77	91	94	95	96	95	97	97

[1] Number of discarded AFP cases per 100,000 children under 15 years of age.
[2] Percent with 2 specimens, 24 hours apart and within 14 days of paralysis onset.
[3] 2005 to 2007 result reported within 28 days and 2008 onwards result reported within 14 days of sample received at laboratory.

Figure 5: Non-polio AFP rate by district, 2015

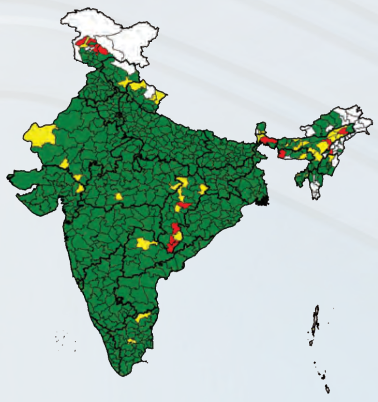

Figure 6: Adequate stool specimen collection percentage by district, 2015

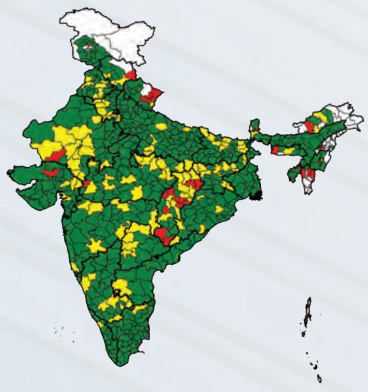

Table 5: Reported cases of vaccine preventable disease, 2006–2015

Year	Polio	Diphtheria	Pertussis	Neonatal Tetanus (% of all Tetanus)	Measles	Rubella	Mumps	Japanese Encephalitis	Congenital Rubella Syndrome
2006	676	2 834	30 088	625 (22%)	64 185	ND	ND	ND	ND
2007	874	3 812	46 674	1 076 (14%)	41 144	ND	ND	4 017	ND
2008	559	3 977	43 697	876 (30%)	44 258	ND	ND	427	ND
2009	741[a]	3 529	60 385	898 (42%)	56 188	ND	ND	653	ND
2010	42[b]	3 434	40 508	521 (30%)	31 458	ND	ND	555	ND
2011	1[c]	4 233	39 091	734 (26%)	33 634	ND	ND	1 214	ND
2012	0[d]	2 525	44 154	588 (24%)	18 668	1 232	ND	ND	ND
2013	0[e]	3 133	31 089	415 (15%)	13 822	3 698	ND	1 078	ND
2014	0[f]	6 094	46 706	492(10%)	24 977	4 870	ND	1 657	ND
2015	0[g]	2 365	25 206	491 (22%)	25 488	3 252	ND	1 620	ND

[a] Excludes two type 1 VDPV and nineteen type 2 VDPV *[b] Excludes five type 2 VDPV* *[c] Excludes six type 2 VDPV and one type 3 VDPV* *[g] Excludes two type 2 VDPV*
[d] Excludes one type 2 VDPV *[e] Excludes five type 2 VDPV* *[f] Excludes three type 2 VDPV*
Source: WHO/UNICEF JRF and EPI/MOHFW *ND=No data*

Figure 7: MCV1 & MCV2 coverage[1] and measles cases[2], 1980–2015

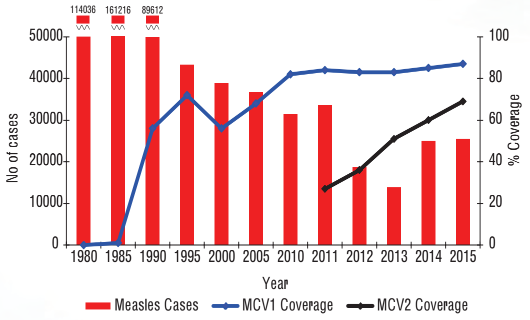

[1] WHO and UNICEF estimates of national immunization coverage, July 2016 revision
[2] WHO vaccine-preventable diseases: monitoring system 2016

Figure 8: MCV1 coverage by province, 2015

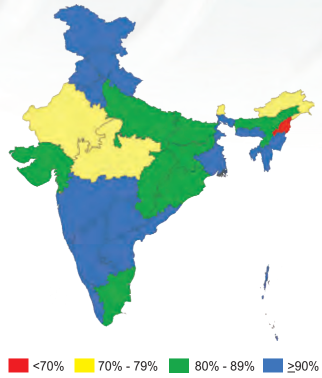

Source: SEAR annual EPI reporting form, 2015 (administrative data)

Table 6: MCV supplementary immunization activities

Year	Vaccine, geographic coverage, target group	Target	Coverage Achieved
2010	M, sub-national, 9 months to 10 years	13 845 686	87%
2011	M, sub-national, 9 months to 10 years	40 167 580	90%
2012	M, sub-national, 9 months to 10 years	76 730 639	92%
2015*	M, sub-national, 1-15 years	890 070	-

*as a part of emergency health response to floods in Tamil Nadu
Source: WHO/UNICEF JRF

Table 7: Provinces with more than 95% MCV1 coverage

Year	Number of districts	%
2010	9	25
2011	24	69
2012	15	43
2013	9	25
2014	14	39
2015	14	39

Source: WHO/UNICEF JRF

Figure 9: Sporadic and outbreak associated measles cases* by month and MR SIA coverage, 2010–2015

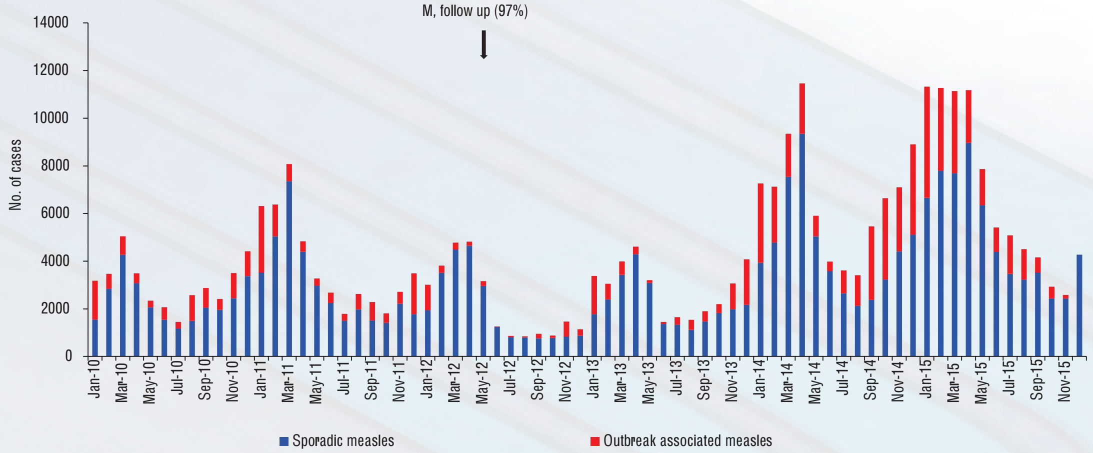

*Includes laboratory confirmed and epidemiologically linked cases
Source: SEAR Monthly VPD reports

Figure 10: Immunity against measles: Immunity profile by age in 2015*

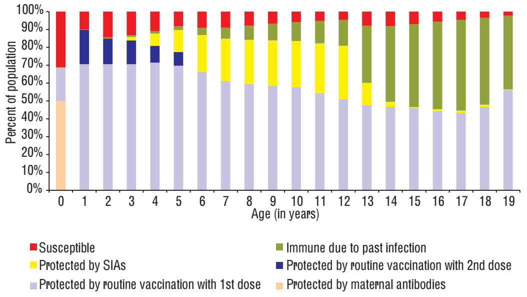

** Modeled using MSP tool ver 2 based on coverage data up to 2015.*

Figure 11: Immunity against measles: Immunity profile by age in 2016*

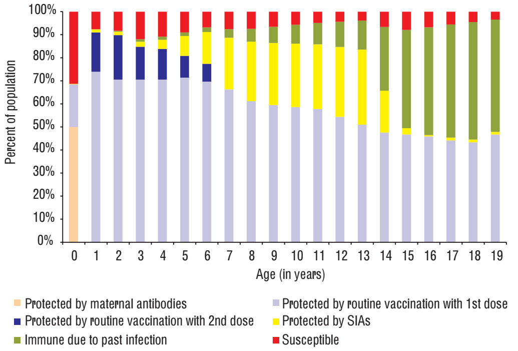

** Modeled using MSP tool ver 2 assuming the schedule and measles containing vaccine (MCV) coverage remain unchanged and MR SIA will be conducted in 10 states of India in 2016.*

Figure 12: Confirmed (Lab and Epi linked) measles outbreak associated cases, by age, 2010–2015

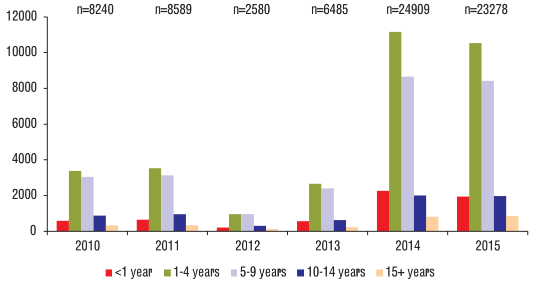

Source: SEAR annual EPI reporting form (multiple years)

Figure 13: Unimmunized confirmed (Lab and Epi linked) measles outbreak associated cases, by age, 2010–2015

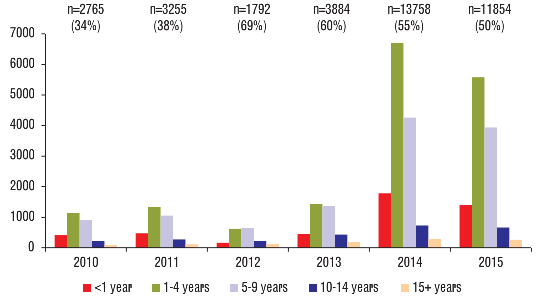

Source: SEAR annual EPI reporting form (multiple years)

Table 8: Suspected sporadic and Outbreak associated measles and rubella cases#, 2010–2015

Year	Routine/sporadic cases				Outbreak associated cases							
	No. of suspected case	No. of death	No. of lab-confirmed measles cases	No. of lab-confirmed rubella cases	No. of suspected outbreak	No. of Outbreak Investigated	No. of case	No. of death	No. of measles outbreak*	No. of confirmed measles case*	No. of confirmed rubella outbreak*	No. of confirmed rubella cases*
2010	27 873	52	–	–	815	242	10 947	40	198	8 984	16	779
2011	35 985	82	–	–	1 085	252	13 987	52	199	10 308	20	1 130
2012	23 705	55	–	–	697	139	6 398	25	72	3 314	26	1 186
2013	26 274	40	–	–	830	339	12 874	24	209	7 858	71	2 734
2014	54 193	110	–	–	1 692	1 012	34 739	101	767	26 044	110	4 993
2015	61 255	202	–	–	2 410	1 321	31 039	187	901	21 771	87	2 451

Source: Monthly VPD Reporting to WHO/SEARO ** Laboratory confirmed & epidemiologically- linked* *ND=No Data*
# 2010 to 2015 only sub-national data, laboratory supported country wide surveillance started from 2015.

Table 9: Quality of field and laboratory surveillance for measles and rubella, 2012–2015

Year	No. of Suspected Measles	Case classification (number)						Indicators					
		Measles			Rubella		Discarded non-measles non-rubella cases	Annual incidence of confirmed Measles cases per million total population	Annual incidence of confirmed Rubella cases per million total population	Proportion of all suspected measles and rubella cases that have had an adequate investigation initiated within 48 hours of notification	Discarded non-measles non-rubella incidence per 100 000 total population	Proportion of subnational administrative units reporting at least two discarded non-measles non-rubella cases per 100 000 total population	Proportion of sub-national surveillance units reporting to the national level on time
		Lab-confirmed	Epi-Linked	Clinically-confirmed	Lab-confirmed	Epi-Linked							
Target →								–	–	80%	2	80%	80%
2012	23 705	303	3 019	ND	114	1 089	1 837	4.62	1.67	ND	2.55	ND	93.64
2013	26 274	910	6 973	ND	373	2 420	1 644	7.22	2.56	ND	1.51	ND	93.93
2014	53 216	3 345	23 730	ND	497	5 344	3 198	21.50	4.64	ND	0.25	ND	94.88
2015	61 255	4 144	20 346	ND	444	2 908	3 565	18.87	2.58	ND	0.27	ND	92.23

Source: SEAR Annual EPI Reporting Form, 2015 *ND=No data*

Table 10: Performance of Laboratory Surveillance, 2012–2015

Year	% Serum specimen collected from suspected measles cases	Total Serum Specimen received in Laboratory	% serum specimens tested	Specimen Positive for Measles IgM		Specimen Positive for Rubella IgM		% Results within 4 of receipt	% Outbreak tested for viral detection	Genotypes detected	
				No.	%	No.	%			Measles	Rubella
2012	3.48	826	99.52	346	42.09	127	26.57	34.62	1.44	D8	ND
2013	5.80	1 525	99.80	720	47.31	352	43.89	49.18	2.36	D8	2B
2014	8.41	4 477	99.73	2 864	63.97	494	30.63	62.00	ND	D4,D8,B3	ND
2015	11.88	7 279	100.00	4 770	65.65	656	26.28	71.00	ND	D4,D8,B3	ND

Source: SEAR Annual EPI Reporting Form, 2015 *ND=No data*

Figure 14: Network of WHO supported surveillance medical officers and laboratories, 2015

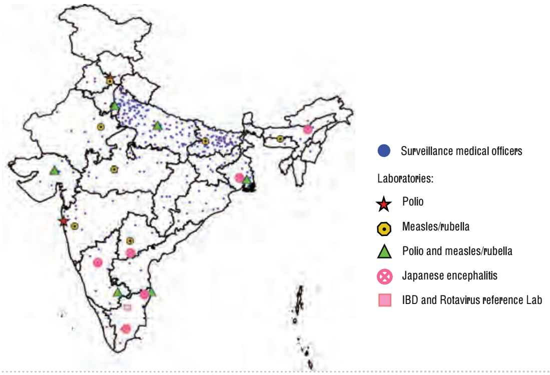

For contact or feedback:
Immunization Division
Ministry of Health and Family Welfare (MOHFW), New Delhi, India
Tel/Fax : +91-11-23062728, Email: pradeephaldar@yahoo.co.in
www.mohfw.nic.in

Immunization and Vaccine Development (IVD)
WHO-SEARO, IP Estate, MG Marg, New Delhi 110002, India
Tel: +91 11 23370804, Fax: +91 11 23370251
Email: SearEpidata@who.int, www.searo.who.int/entity/immunization
